# Civil servants’ integrity in public sector: the case of Nepal

**DOI:** 10.1016/j.heliyon.2022.e12632

**Published:** 2022-12-24

**Authors:** Rajan Khanal, Anil Kumar Gupta, Prakash C. Bhattarai

**Affiliations:** a Nepal Administrative Staff College, Jawalakhel Lalitpur, Nepal; b Kathmandu University-School of Education, Lalitpur, Nepal

**Keywords:** Civil servant, Citizen, Integrity, Public service, Nepal

## Abstract

Civil servants' integrity in delivering public service is the heart and soul of public sector governance worldwide, including in Nepal. Ensuring a higher level of integrity for civil servants is complex, as it is affected by several factors. With this consideration, this study aims to examine the factors affecting the integrity of civil servants in the delivery of public services in Nepal. Data on this subject were derived from the Nepal National Governance Survey 2017/18, and analyzed using a logistic regression model. The findings revealed that citizens perceived civil servants’ integrity in public service was positively affected by civil servants’ compliance with rules, their responsiveness in delivering service, their service on time, their hassle-free service, and their adequate salary, whereas negatively affected by civil servants’ prior network/connection with citizens and their asking or receiving a bribe for public service. Therefore, the concerned authorities should focus on these factors to build and maintain civil servants' integrity in delivering public service. Our findings provide empirical evidence for concerned authorities who can contribute to adopting innovative governance approaches and appropriate policies to build and maintain civil servants’ integrity in the coming days. The study contributes to the field of integrity and public service by highlighting the factors affecting the integrity of civil servants in the delivery of public services.

## 1 Introduction

Over the last two decades, governance, ethics, and integrity have piqued the interest of academics, public service practitioners, politicians, media, and general public (Kolthoff et al., 2013). Everyone has an interest and a role in maintaining the integrity of government in society (McLeod et al., 2022). The government has recently devoted much attention to civil servants' integrity in delivering public services. In a democratic society, public service integrity lies in behaving under the values of giving importance to its citizens-referred to as ‘citizen administrator value congruence’ (Huberts, 2014). In practice, civil servants are supposed to demonstrate integrity in delivering public service, and thus they are expected to be capable and proficient in providing public services. They are also considered ethically competent and with a strong nous of right and wrong.

Civil servants’ actions in delivering public services are determined by their moral or ethical values and standards. Ethical values and standards positively motivate civil servants to demonstrate their integrity. Service users also expect civil servants to deliver public service in their best interests with maintaining integrity, civility, impartiality, care, diligence, and honesty. Their ethical expectations are rising daily (Adeyemi, 2016). However, there has been growing concern that the integrity standard is declining (Heywood et al., 2017). Lowering the standard of integrity violates ethical and moral values, which creates a favorable atmosphere for unethical behavior. Unethical behavior negatively affects efficient and effective public services (Adeyemi, 2016). Therefore, ethical behavior in public office is essential because violations of these shared moral norms cast public office into contempt (Jenkins, 2015). Integrity codes, rules, and procedures should be adequately communicated within the organization to be effective (Hoekstra et al., 2022).

Demonstrating integrity in delivering public service is not easy but full of challenges. Civil servants provide public service with a public service ethos to address the challenge. Public service ethos is a set of values that includes honesty, integrity, accountability, and probity (Lawton et al., 2013). Upholding the public service ethos is an immense quality and a responsibility of civil servants, which helps increase citizens’ trust in public service. Practices of public service ethos in delivering public service are decisive for ensuring integrity and earning public trust. Therefore, civil servants, who are directly involved in providing public services, are aware of the public service ethos, and put it into practice. Practicing public service ethos such as honesty, fairness, respect, loyalty, sincerity, and impartiality ensures a high level of integrity in civil servants, and thus the integrity of civil servants is unthinkable without practices of this ethos. Therefore, individuals (civil servants) working in public services are bound, subscribed to, and inspired by a public service ethos (Lawton 1998; Vandenabeele et al., 2006). Here practices of public service ethos can be institutionalized for ensuring civil servants’ integrity.

The government of Nepal has paid much attention to public service ethos in delivering public services since the establishment of democracy in 1951. Since then, many reform commissions, constitutional bodies, state institutions, laws, policies, guidelines, and directives have been formed over the years (Paudel and Gupta, 2019a) to put public service ethos into practice. However, evidence shows that the organizational culture for practicing public service ethos seems low. The Nepali civil service is accused of being dysfunctional, fragmented, poorly organized, inefficient, ineffective, evasive, buck-passing, delayed, reckless, self-seeking, inexplicable, unaccountable, corrupt, non-transparent, irresponsible, and unable to meet citizens' expectations (see: Dangal, 2005; Shakya, 2009; Bajpai, 2014; Bhattarai, 2017; Gupta, 2018; Paudel and Gupta, 2019b; Lamsal and Gupta, 2022; Bhattarai and Gupta, 2022). To address these issues, the Constitution of Nepal (2015) has envisioned making public administration fair, competent, impartial, transparent, free of corruption, accountable, and participatory (GoN, 2015, p.30). Likewise, the Government of Nepal has included in its policy and program (2020/2021) a provision to take regular oath as part of the organizational culture to make civil servants more honest and ethical in their work. The oath is ‘I will not engage in corruption, I will prevent corruption, and I will work honestly for my country and people’ (GoN, 2020, p. 57). Institutionalizing such an oath-taking system as part of the organizational culture can be seen as a good to make civil servants honest and ethical in delivering public services (Bhattarai and Gupta, 2022), but this has not yet been put into practice.

Many scholars carried out studies on unethical behavior, ethics, corruption, codes of conduct, public integrity, governments’ integrity, integrity management, integrity system, and integrity violation (see: Grundstein-Amado, 2001; Balogun, 2003; Walker, 2005; Bhattarai, 2009; Briggs, 2009; Shakya, 2009; Hoekstra and Kaptein, 2012; Elcock, 2012; Lawton et al., 2013; Haruna, 2008; Huberts, 2014, 2018; Hubert and Van Montfort, 2020; Ertas, 2021; Khadka and Bhattarai, 2021; Hoekstra et al., 2022; Huberts et al., 2022; Neupane et al., 2022). The studies provide different perspectives and theoretical frameworks to understand public service values and ethos. However, the authors hardly found empirical research on the factors influencing civil servants’ integrity in delivering public service. There is not much known about how civil servants’ compliance with rules, their responsiveness, their delivery of services on time, their providing of hassle-free service, their prior network/connection with citizens, their inadequate current salary, and their acceptance or asking for bribes affect integrity in public service delivery. With this consideration, this study aimed to examine the factors affecting the integrity of civil servants in the delivery of public service, using the Nepal National Governance Survey 2017/18 data set. In this favor, this study proposes the following hypotheses to support the research objective.

• Civil servants who follow the rules in delivering public services demonstrate a higher level of integrity.
• Civil servants who are responsive in delivering public services demonstrate a higher level of integrity.
• Civil servants who provide services on time demonstrate a higher level of integrity.
• Civil servants who provide hassle-free public services to citizens demonstrate a higher level of integrity.
• The preference of civil servants for citizens they know personally (prior network/connection) in delivering public services a lower level of integrity.
• Civil servants who ask or accept bribes for public services show a lower level of integrity.

The result of the hypotheses generates empirical evidence, bridge knowledge gaps, and spark scholarly discussion about the integrity of civil servants in the delivery of public services in Nepal. The study implied that factors affecting the integrity of civil servants should be a strategic concern for policy makers, politicians, and practitioners to adopt better strategies and initiatives in the coming days. This study also seeks to offer insights for rethinking policy and governance approaches to ensure higher levels of integrity of civil servants in delivering public services in changing socio-political and administrative contexts. In presenting the insights, literature on integrity, including its Western and Eastern traditions, has been presented in the following section.

## 2 Literature review

### 2.1 Integrity: what does it mean?

The notion of integrity has acquired prominence in governance and administration literature. While integrity has become a central concept and topic in government and governance research and actual policy making at all levels, multiple perspectives and interpretations exist (Huberts et al., 2022). Integrity is a value-laden concept (Haruna, 2008), and has no perfect universally agreed definition (Cox et al., 2008; cited in Brillantes and Fernandez, 2011). Many scholars define integrity as wholeness or consistency, while others define it as synonymous with specific values such as honesty, reliability, or incorruptibility (Huberts et al., 2022). Montefiore and Vines (1999) state that integrity is a sense of completeness, wholeness, coherence, and consistency of principles and values. In the same vein, Heywood et al. (2017) argued that integrity is not just being not corrupt, and involves wholeness, consistent action with principles, morality, and process. In the view of Huberts (2014), integrity is the quality of behavior in conformity with applicable moral principles, standards, and rules. Hubert and Van Montfort (2020) consider integrity as acting by relevant moral and legal values and norms. Acting with integrity is identical to acting ethically or morally (De George, 1993). Integrity is also seen as the attribute of being honest and truthful (Amundsen and Pinto De Andrade, 2009). Armstrong (2005) argues that integrity is honesty and trustworthiness in executing official tasks. International standards of integrity for civil servants include principles of honesty, fairness, transparency, accountability, impartiality, prudent use of state resources, and non-discrimination, all of which are essential for the proper functioning of the public service and for ensuring citizens' trust (Maisuradze et al., 2022).

Most scholars focus on honesty and trustworthiness in the definition mentioned above. Is integrity just simply honesty (Trevino et al., 2000)? Carter (1996) believes that integrity goes beyond honesty and being willing to behave openly and knowingly, following right instead of wrong based on moral contemplation. Dunn (2009) argued that integrity needs more than just honesty. In addition, he stated that, in reading the literature on organizational behavior, especially empirical studies on integrity, honesty might be concluded as a synonym for integrity. Integrity is used interchangeably with honesty since honesty indicates morality, ethics, impartiality, fairness, and truthfulness. Personal integrity implies honesty and coherence to the self (Grundstein-Amado, 2001). Integrity is the trait of being honest, truthful, kind, whole, and one. The U.N. Committee of Experts on Public Administration defines integrity as “the honesty and reliability of civil servants in executing their tasks” (as quoted in D'Alterio, 2017). Integrity concerns the honesty of civil servants’ behavior. Based on scholars’ ideas, this paper defines integrity as the honesty of civil servants in delivering public services. An honest civil servant provides public service with moral and ethical values.

### 2.2 Integrity in western and eastern tradition

Integrity has long piqued the interest of philosophy and the world’s wisdom because of its connection with ethics (Miller, 2017). Ethics and integrity have been interpreted in various ways in western and eastern traditions.

#### 2.2.1 Western tradition

Bentham and Mills’ utilitarianism, Kantian deontology ethics, and Aristotelian virtue ethics are famous in the western tradition. Each provides a normative framework for specifying what people should do (Morrell and Dahlmann, 2022). Utilitarianism, often known as consequentialism, is a moral theory that argues that morally good action yields satisfaction and pleasure. Utilitarianism determines morality based on the outcome of the intervention, and states that the moral course maximizes value over non-value and seeks the most significant benefit for the greatest number of people (Vearrier and Henderson, 2021). Whether an action is right or wrong depends on the ability of that action to maximize positive outcomes (Tseng and Wang, 2021). An action is deemed ethical if it has more positive consequences than negative ones. It states that an action is moral if it maximizes the happiness of many individuals (Tzoanou, 2013), suggesting that harm to some may be acceptable concerning the overall net benefit to the group as a whole (Vearrier and Henderson, 2021). An action is morally justifiable if it maximizes many people’s happiness and minimizes their harm (Lawton et al., 2013). Therefore, individuals choose activities that maximize overall utility or satisfaction (Amundsen and Pinto De Andrade, 2009). With this viewpoint, civil servants would act and perform to maximize the happiness and well-being of the state and its citizens. Civil servants’ actions and behaviors rely on their effects, which are ethically and morally wrong or right. It is ethically correct if the product maximizes the state's and citizens' happiness and satisfaction.

Kantian deontology ethics, commonly known as ‘duty ethics,’ believe in ethical standards and emphasize the wrongness or rightness of behavior and actions rather than the rightness or wrongness of their consequences (Amundsen and Pinto De Andrade, 2009). People have to abide by their moral duty or obligation rather than the consequences of their actions (Tseng and Wang, 2021). Deontological ethics evaluates actions concerning duties and obligations (Yuan et al., 2022). The morality of action resides solely in fulfilling one's duty (Saleh et al., 2022). Kant grounded his duty-based ethics on universal moral obligations (Tseng and Wang, 2021), and an individual's moral or ethical duty is to comply with universal moral rules (Tzoanou, 2013). The ethics of duties convey morality through universal laws in all contexts. Kant believed that an action is ethically and morally commendable when performed for a sense of duty rather than self-interest (Amundsen and Pinto De Andrade, 2009; Lawton et al., 2013). Kant’s view of duty is similar to the concept of ‘karma’ (people have the right to do their duty without expectation) proposed in the Bhagavad Gita (holy writings of Hinduism). Bhagavad Gita intends that ‘duty is vital’. Both Bhagavad Gita and Kant caution against the rule of the senses, and agree that the moral law is to perform one’s duty for the sake of doing, without any personal attachment or hope of reward (Radakrishnan, 1911). Kantian deontology ethics, ethics moral credit is granted to civil servants not only because they act and perform morally correct behaviours, but also because it is their duty. Civil servants should be governed by moral duty and have the right motive for taking the right action for the right reason. Moral duty may serve as the sole motive for the ethical act of civil servants. Civil servants’ efforts are only ethically noble if they are governed by duty and a sense of just and fairness instead of self-interest.

Virtue is central to Aristotelian virtue ethics and remains one of the most influential theories in Western moral philosophy. Socrates’ and Plato’s ideas about virtues influenced Aristotle, who followed them and considered them the center of a good life (Papouli, 2019). Virtue was at the heart of most previous Socratic dialogues (Kremm, 2009). In Plato’s early dialogues, Socrates argues that knowing good and evil is adequate to be a fair, courageous, and humble person, but for Aristotle, being a decent person entails more than just knowing what is right and wrong, believing that one cannot be genuinely wise unless one has a good character (Devereux, 1992). Aristotelian virtue ethics focuses on people’s moral character, moral intent, and exercise of virtue (Kraut, 2006; Vardy and Grosch, 1999). Aristotle remarks that virtues are good habits of heart and mind that are essential to developing and maintaining good moral character and behaviour (Aristotle, 2004). A virtue must be seen in action to be a virtue (Fowers et al., 2022), and a virtuous moral actor exercises relevant virtues in appropriate circumstances (Bellazzi and Boyneburgk, 2020). Virtuous moral actors respond in the right way at the right time, to the right thing, to the right people, and with the right intentions (Carr, 2003). Under this viewpoint, civil servants should have moral character, good intent, and practice virtues while doing their duties. Civil servants’ actions are not virtuous unless they are engaged in developing moral character and have a solid aspiration to do what is right. Therefore, civil servants should be highly relied on acquiring and developing a virtuous character through the exercise of moral virtues, which leads civil servants to act in the right and appropriate way for the nation and citizens. Aristotle’s virtue ethics encourage civil servants to develop moral character and proper sense for doing morally and ethically and becoming better for society and nation.

#### 2.2.2 Eastern tradition

In eastern tradition, ethics and integrity are reflected in unique ways. Many eastern philosophers defined ethics and integrity; however, this part only discusses Confucius’ and Chanakyas’ viewpoints. Confucius and Kautilya were both creative intellectuals who argued for establishing moral order and an ethical environment (Sihag, 2016). In the Confucian view, duties, virtues, and morals diverge from western ethics (Woods and Lamond, 2011). Confucius offers a system of virtue ethics (Chan, 2003; Wang, 2006), and exercise of the virtues are rudiments for being ‘human’ (Sundararajan, 2005). Confucius encourages humans to live frugally and humbly (Huo and Kristjánsson, 2020). Confucius emphasizes caring for others with kindness and governing a state with ethics, which reflects the importance of moral principles in politics (Hu et al., 2021). Confucian ethics views the individual self as an integral node in the network of social relationships, and thus the process of self-cultivation to become a good leader is inevitably facilitated by a socially sanctioned understanding of the highest good (Yuan et al., 2022). Confucian ideology focuses on the self-cultivation of virtues and the upkeep of personal ethics (Gupte, 2018). Confucius stated that man/women’s existence rests in his/her integrity, and a man/woman without integrity can exist only through luck, and a leader should erect and act with integrity to lead people effectively (as cited in Ang and Low, 2012). Confucius also said, ‘When you encounter a gentleman (woman) of virtue, learn from them; when you meet a person without virtue, examine yourself to see if you have the same deficiencies as he/she has’ (as quoted in Lau, 1979, p.74). Confucian virtue ethics is not only concerned with cultivating individual virtues, but instead believes that progress in the cultivation of individual virtues gradually leads to desired social outcomes, namely, families are well regulated, states are well governed, and, therefore, the collective harmonization of wills can be achieved (Yuan et al., 2022). Under Confucius’s viewpoint, civil servants should pose and positively act with integrity in their professional, family, and social lives. Civil servants should have virtue and live and lead in the right way. For this, civil servants should self-cultivate, and have self-discipline and self-growth, which hone their moral and ethical character. Confucian virtue ethics such as benevolence, righteousness and ritual propriety, loyalty, reliability, and wisdom can help prevent unethical behavior among civil servants.

Kautilya, also known as Chanakya, discusses virtue ethics and action-oriented ethical principles (Gupte, 2018). Moral values, as per Kautilya, are a means of gaining success in this life and opening the route to heaven after death (Sihag, 2007). Kautilya viewed virtues and morals as embedded in good practices, and dharma is crucial to creating an ethical climate inside the state and at the individual level to ensure productivity (Gupte, 2018). Kautilya claimed that creating an ethical environment in the state is necessary for maintaining law and order (Sihag, 2016; Basu and Miroshnik, 2021), and is dependent mainly on excellent ethical conduct obtained via self-discipline and ethical anchoring (Gupte, 2018). Kautilya highlighted the relevance of moral philosophy in understanding the difference between moral and immoral and between good and evil (Sihag, 2005). Kautilya believed that bureaucratic organizations are necessary to facilitate public service, but bureaucrats may accept corrupt resources in delivering the services, which is often hard to detect (Basu and Miroshnik, 2021). That may be why Kautilya argued for a fair, clean, and caring administration. According to the viewpoint of Kautaliya, civil servants should have moral values, adhere to their dharma, and be responsible for creating an ethical climate. They should be entirely devoted to their moral duties, which should be fair and just. Civil servants should be made accountable for their moral responsibilities and be disciplined and rewarded based on the quality of their work. Civil servants should be bound with good ethical conduct to maintain society’s law, order, and prosperity. They should have no self-interest, and their pleasure should be based on the well-being of the citizens they serve.

The summary of the literature review is presented in Table 1.

**Table 1:** Summary of literature.

Concept	Main Ideas	Sources
Integrity	• Value-laden concept • Sense of completeness, wholeness, coherence, and consistency • Not just being not corrupt and involves wholeness consistent action with principles, morality, and process. • Acting in accordance with relevant moral and legal values and norms • Attribute of being honest and truthful • honesty and trustworthiness in executing official tasks	Montefiore and Vines (1999); Armstrong (2005); Haruna (2008); Amundsen and Pinto De Andrade (2009); Heywood et al. (2017); Hubert and Van Montfort (2020)
Utilitarianism	• An action is morally justifiable if it maximizes many people’s happiness and minimizes their harm • Action is right or wrong depends on whether that action can maximize positive outcomes • Maximizes value over non-value and seeks the greatest benefit for the greatest number of people	Lawton et al. (2013); Vearrier and Henderson (2021); Tseng and Wang (2021)
Kantian deontology ethics	• Emphasize the wrongness or rightness of behavior and actions rather than the rightness or wrongness of their consequences • Evaluate actions by loci to duty and obligation • Comply universal moral rules • Action is ethically and morally commendable when it is performed for a sense of duty than self-interest	Amundsen and Pinto De Andrade (2009); Tzoanou (2013); Lawton et al. (2013); Yuan et al. (2022)
Aristotelian virtue ethics	• Virtue ethics focuses on people’s moral character, moral intent, and exercise of virtue • Being a decent person entails more than just knowing what is right and wrong, and have a good character • Virtue must be seen in action to be a virtue • Virtuous moral actor exercises relevant virtues in appropriate circumstances	Devereux (1992); Vardy and Grosch (1999); Kraut (2006); Bellazzi and Boyneburgk (2020); Fowers et al. (2022)
Confucius virtue ethics	• Focus on the self-cultivation of virtues and the upkeep of personal ethics • Exercise of the virtues are rudiments for being ‘human’ • Caring for others with kindness and governing a state with ethics • Progress in the cultivation of individual virtues gradually leads to desired social outcomes	Sundararajan, 2005; Gupte (2018); Hu et al. (2021); Yuan et al. (2022)
Kautilya virtue ethics	• Virtues and morals as embedded in good practices • Dharma is crucial to creating an ethical climate inside the state and at the individual level to ensure productivity • Ethical environment in the state is necessary for maintaining law and order • Emphasized fair, clean, and caring administration	Sihag (2016); Gupte (2018); Basu and Miroshnik (2021)

## 3 Study methods

### 3.1 Data

This paper’s analysis is derived from the quantitative data extracted from the nationally representative Nepal National Governance Survey (NNGS) 2017/18. The survey was administered by Nepal Administrative Staff College (NASC) between December 2017 and March 2018 to capture the experience of Nepali citizens on different aspects of state governance. By utilizing a multistage cluster sampling design, this survey sampled 12,920 individual respondents aged 18 and above, representing all seven provinces and three ecological zones. Trained enumerators conducted face-to-face interviews, yielding 12,872 filled-up questionnaires with a 99.6% response rate (NASC, 2018). The survey consists of three broad governance dimensions: the foundation of governance, the infrastructure of governance, and service delivery (Table 2).

**Table 2:** Dimension of governance.

Foundations of Governance	Infrastructure of Governance	Service Delivery
Voice and participation; integrity and accountability; justice and social inclusion; and the rule of law	Elections; constitution and constitutional provisions; realization of constitutional freedoms; provincial and local government; political institutions; social association and civic awareness; information and communication and security and protection	Experience of public service; support for receiving services; attempts made to receive public services; source of information about services; service fee and additional expenses; public service environment

Source: NNGS, 2017/18, p.3.

### 3.2 Study variables: dependent and independent

Civil servants’ integrity in public service delivery was used as a dependent variable. Under the heading of integrity and accountability, this survey collected information on civil servants’ integrity by asking, “how honest do you think civil servants are in their work?”. This question had four answers: very honest, honest to some extent, not honest, and don’t know/can’t say. This study dropped the don’t know/can’t say option as it does not provide a clear-cut answer. Out of 12872 respondents, 487 were answered can’t say/don’t know; hence, the sample size for the analysis became 12385. For this study, ‘civil servants’ honesty’ is used as a dependent variable, referred to as ‘integrity’. The dependent variable honesty of civil servants is perception-based. This study used the perception-based honesty index, because this survey gathered data on citizens’ perceptions of the honesty of civil servants. This study used seven independent variables (civil servants’ compliance with rules, responsiveness, delivering service on time, providing hassle-free service, prior network/connection with citizens, having an adequate salary, and accepting/asking for a bribe) to examine the effect on the dependent variable (civil servants’ integrity) with or without statistical significance (see: Tables 3 and 4).

**Table 3:** Study variables.

Dependent	Independent
Integrity of civil servants	Civil servants’ compliance with rules
Independent	Responsiveness
	Service on time
	Hassle-free service
	Prior network/connection with citizens
	Salary
	Asking/accepting bribe

**Table 4:** Coding of study variables.

Variable	Survey statements	Coding
Dependent		
Integrity of civil servants	How honest do you think civil servants are in their work? [options: 1 = very honest, 2 = honest to some extent, 3 = not very honest, 4 = not honest at all, 99 = don’t know/can’t say]	Very honest and honest to some extent were merged and labelled as ‘being integrity’, with the code of ‘1’ and not honest at all was labelled as ‘not being integrity’, with the code of ‘0’. Don’t know/can’t say was omitted.
Independent		
Civil servants’ compliance with rules	To what extent do you agree that the government employees follow the rules? [Options: 1 = strongly agree, 2 = agree to some extent, 3 = disagree to some extent, 4 = strongly disagree, 99 = don’t know/can’t say]	Strongly agree and agree to some extent were merged and labelled as ‘yes’ coded as ‘1’ while disagree to some extent and strongly disagree merged and labelled as ‘no’ coded as ‘0’. Don’t know/can’t say was omitted.
Responsiveness	To what extent do you agree that: a The government employees properly listen to and understand concerns of service recipients. b. The government employees provide clear information about the service. c. The government employees use polite language. d. The government employees behave in a friendly/cordial manner. [Options: 1 = strongly agree, 2 = agree to some extent, 3 = disagree to some extent, 4 = strongly disagree, 99 = don’t know/can’t say]	The responsiveness of civil servants was measured with the four statements of the survey. Strongly agree and agree to some extent were merged and labelled as ‘yes’ coded as ‘1’ while disagree to some extent and strongly disagree merged and labelled as ‘no’ coded as ‘0’. Don’t know/can’t say was omitted.
Service on time	To what extent do you agree that the government employees provide service in time? [Options: 1 = strongly agree, 2 = agree to some extent, 3 = disagree to some extent, 4 = strongly disagree, 99 = don’t know/can’t say]	Strongly agree and agree to some extent were merged and labelled as ‘yes’ coded as ‘1’ while disagree to some extent and strongly disagree merged and labelled as ‘no’ coded as ‘0’. Don’t know/can’t say was omitted.
Hassle-free service	To what extent do you agree that the government employees do not create any hassles/problems? [Options: 1 = strongly agree, 2 = agree to some extent, 3 = disagree to some extent, 4 = strongly disagree, 99 = don’t know/can’t say]	Strongly agree and agree to some extent were merged and labelled as ‘yes’ coded as ‘1’ while disagree to some extent and strongly disagree merged and labelled as ‘no’ coded as ‘0’. Don’t know/can’t say was omitted.
Prior network/connection with citizens	To what extent do you agree that if one has connections/networks, work is done in any way? [Options: 1 = strongly agree, 2 = agree to some extent, 3 = disagree to some extent, 4 = strongly disagree, 99 = don’t know/can’t say]	Strongly agree and agree to some extent were merged and labelled as ‘yes’ coded as ‘1’ while disagree to some extent and strongly disagree merged and labelled as ‘no’ coded as ‘0’. Don’t know/can’t say was omitted.
Salary	To what extent do you agree that government employees receive sufficient salary and facilities? [Options: 1 = strongly agree, 2 = agree to some extent, 3 = disagree to some extent, 4 = strongly disagree, 99 = don’t know/can’t say]	Strongly agree and agree to some extent were merged and labelled as ‘yes’ coded as ‘1’ while disagree to some extent and strongly disagree merged and labelled as ‘no’ coded as ‘0’. Don’t know/can’t say was omitted.
Asking/accepting bribe	In the last 12 months, did you or your family member pay or was asked to pay a bribe while accessing any public service? [Options: 1 = yes, 2 = no, 3 = did not receive any public service, 99 = don’t know/can’t say]	Paid/asked bribe is labelled as ‘yes’ coded as ‘1’ while not paid/asked bribe is labelled as ‘no’ coded as ‘0’. Did not receive any public service, and don’t know/can’t say were omitted.

### 3.3 Logistic regression

When the dependent variable is dichotomous and the independent variable is continuous, categorical, or both, binary logistic regression is generally used (Park, 2013; Jawa, 2022). In this study, a binary logistic regression model was used to examine the effect of independent variables on the dependent variable, as the dependent and independent variables were coded with binary options. The dependent variable ‘civil servants’ integrity’ was classified into two groups: a) ‘1’ = being integrity and b) ‘0’ = not being integrity. Like the dependent variable, all independent variables were categorized into two parts. The first category was coded as ‘1’ for following rules during service delivery, being responsive in service delivery, delivering service on time, delivering hassle-free service, having a prior connection/network with the citizen, having an adequate salary, and accepting/asking bribes from the service user. The second category was coded as ‘0’ for not following rules during service delivery, not being responsive in service delivery, not delivering service on time, creating hassle in service, not having a prior connection/network with the service user, not having a sufficient salary, and not ask bribe with the service user (see: Table 4).

The binary logistic regression equation model:

(1) $$Logit\;\left(y\right)=\ln\left\{\frac{P}{1-P}\right\}=\alpha +\beta_{1}k_{1}+\beta_{2}k_{2}+\beta_{3}k_{3}+\beta_{4}k_{4}\ldots .+\beta nkn$$

Where, ‘P’ is the probability of demonstrating integrity; ‘(1-P)’ is the probability of not demonstrating integrity; ‘P/(1-P)’ is the odds ratio; ‘α’ is the ‘y’ intercept; ‘β’ is regression coefficients; ‘k’ = independent variables (compliance with rule, responsiveness, on-time service delivery, hassle-free service, prior network/connection, salary, and asked bribe).

All necessary conditions of the binary logistic regression model were met, and the test was carried out using the SPSS software 26 version. For data interpretation, the odds ratio was used. The generated data and information were organized, tabulated, and logically interpreted afterwards using public service and integrity literature.

## 4 Findings

A binary logistic regression was run with seven independent and dependent variables (Table 5). In this model, civil servants’ compliance with rules, their responsiveness, their delivery of service on time, providing hassle-free service, prior network/connection with citizens, having an adequate salary, and accepting/asking for bribes were included as independent variables, and civil servants’ integrity was included as a dependent variable. The odds ratio (OR) yielded several impressive results. Compliance with rule has a significant positive impact on civil servants’ integrity (OR = 1.433, p < .001). Civil servants who follow the rules while delivering public services are 143.3 percentage points more likely to demonstrate integrity than those who do not. Responsiveness and civil servants’ integrity are significantly positively associated (OR = 1.390, p < .001). Civil servants who listen, inform, and respond to citizen concerns while delivering public services are 139 percentage points more likely to demonstrate integrity than those who do not.

**Table 5:** Coefficients of binary logistic regression for civil servants’ integrity in public service.

Independent Variables	B	S.E.	Odd Ratio
Compliance with rule	.359	.066	1.433∗
Responsiveness	.329	.076	1.390∗
Timely Service Delivery	.179	.074	1.196∗∗
Hassle-free Service	.910	.050	2.485∗
Network/connection	-.435	.076	.648∗
Salary	.115	.055	1.121∗∗
Asked Bribe	-1.002	.073	.367∗
Constant	.265	.085	1.303

Nagelkerke R Square = .169, ∗p = 0.00, ∗∗p < 0.05.

Service delivery on time, like compliance with rules and responsiveness, has a significant positive impact on civil servants’ integrity (OR = 1.196, p < 005). Civil servants who deliver public services on time are 119.6 percentage points more likely to demonstrate integrity. The impact of hassle-free service on civil servants’ integrity is massive and significantly positive (OR = 2.485, p < .001), implying that civil servants who deliver hassle-free public services are 248.5 percentage points more likely to demonstrate integrity. A significantly positive association is also detected between salary and civil servants’ integrity (OR = 1.121, p < .005), indicating that civil servants who are paid adequately are 112.1 percentage points more likely to demonstrate integrity. This situation, conversely, is exclusively different for network/connection and accepting/asking for bribes.

The impact of network/connection and accepting/asking appears to be negative on civil servants’ integrity. The value of OR is .648 for network/connection, which is significant at p < .001. The result suggests that civil servants who deliver high-quality public services for the citizens they know personally in a friendly/cordial manner are 64.8 percentage points less likely to demonstrate integrity. Similarly, civil servants who accept/ask a bribe to deliver public services are 36.7 percentage points less likely to display integrity, indicating a negative effect on civil servants’ integrity. Nagelkerke R Square statistics of compliance with rules, responsiveness, delivering service on time, providing hassle-free service, prior network/connection with citizens, having an adequate salary, and accepting/asking for bribes explain 16.9 percent of the variation in civil servants’ integrity.

## 5 Discussion

Civil servants deliver public services on behalf of the state to citizens. Citizens increasingly expect civil servants to deliver public service with high ethics and integrity. Civil servants’ integrity in providing public service is affected by several factors, which are discussed below.

### 5.1 Compliance with rule

Integrity denotes consistency between rules and behaviour, and is required to understand the spirit of the law and behave accordingly (Kaptein, 2014). Integrity emphasizes ‘lawfulness-acting in following the rules and norms of the law’ (Huberts et al., 2022, p. 330). Compliance positively affects civil servants’ integrity in delivering public service. As confirmed by this study, adhering to predetermined rules and regulations in providing public services fosters civil servants’ integrity. This may be because Nepal's civil servants (bureaucrats) follow rules, regulations, and procedures in performing their duties (Gupta et al., 2019). Therefore, the action and performance of civil servants should be bound by pre-defined rules and standards (Paudel and Gupta, 2019a). Breach of formal and pre-defined rules in delivering public service stances is a barrier to integrity. Civil servants need to obey the law in delivering public services to ensure and maintain integrity. It is because rules and regulations control unethical behavior (Lawton et al., 2013). The rules inspire ethical conduct by establishing a minimum standard for moral action (Amundsen and Pinto De Andrade, 2009).

According to NNGS 2017/18, only 15.7 percent of Nepali citizens strongly believe that government employee follows the rules (NASC, 2018). Both citizens and civil servants are involved in breaching rules in public service. In general, citizens violate predefined rules when they are not entitled to receive public services as defined by the rules, and civil servants do not have the right to provide services according to the rules (Bhattarai and Gupta, 2022). Violation of the rules supports unethical behavior and creates a favorable environment for corruption (Lamsal and Gupta, 2022). Civil servants must obey the law to be ethical and demonstrate integrity. In certain circumstances, civil servants adhere to the regulations to legal ramifications rather than to be ethical and honest. Integrity is not implied in rigidly adhering to the rules, but in assuring 'fair play' (Kaiser and Hogan, 2010). Hence, there should be clear rules and procedures, which should be pretty followed in delivering public service. Reasonably adhere pre-defined regulations is an essential prerequisite for maintaining integrity in civil servants.

### 5.2 Responsiveness

Demand for responsive public services has increased over the past year (Lamsal and Gupta, 2022). The subject of responsiveness is an obstinate perennial in the realm of public service ethics (Mulgan, 2008). Responsiveness in public service helps to meet citizens’ real expectations. It entails actively listening, supporting, and responding to citizens and their needs empathically (Pokharel et al., 2018). For Engdaw (2019), responsiveness involves the service provider’s eagerness to the backing service user and deliver the requested services on time. Citizens expect civil servants to be more responsive (Lamsal and Gupta, 2022). As civil servants become more responsive, the likelihood of demonstrating integrity rises. This study indicated that civil servants’ responsiveness to public service positively influences their integrity. Civil servants who listen attentively, provide clear information, gently assist in simplifying the bureaucratic process, demonstrate a high level of integrity. It is because responsive civil servants satisfy citizens' needs and expectations (Gupta, 2018). The delivery of responsive public services assures responsive governance and fosters integrity in civil servants. The absence of responsiveness in civil servants lessens to demonstrate integrity. Responsiveness in delivering public service boosts civil servants’ integrity and ethics. Civil servants, therefore, must take comprehensive measures for proactively providing responsive public services.

### 5.3 On-time service delivery

A state has to provide high-quality goods and services with appropriate quality standards in a timely manner (Pandey, 2019), where public service should be delivered as swiftly as possible. Public institutions guarantee a public service within a given time, and reasons such as administrative ineptitude, corruption, and red-tapism have strongly contributed to the demand for time-limited services (Pareek and Sole, 2020). Citizens have the right to obtain public services within a specified time frame; therefore, civil servants should strictly deliver public services on time. If citizens do not receive service on time, they become dissatisfied, negatively rate the ethics and honesty of civil servants, and raise questions on their integrity. Civil servants should not be delayed in delivering public service unless reasonable cause. This study showed that the timely delivery of public services positively affects civil servants’ integrity. Civil servants who provide public services on time are more likely to demonstrate integrity. Service delivery on time lessens corruption and fosters integrity in civil servants. The government of Nepal has policy provisions for delivering public services on time. The civil service act (1993) mandates that service users be informed about work-related concerns, processes, and time to perform the work (GoN, 1993). The good governance (management and operation) act (2008) attempted to make civil servants ethical and honest with emphasis on timely, accountable, transparent, and corruption-free public services (GoN, 2008). This act has a mechanism for punishing errant civil servants if they fail to provide the service on time.

Similarly, the introduction of the citizen charter promotes timely and corruption-free public service (Gupta and Shrestha, 2021). All procedures and required time to obtain service are written clearly in the citizen charter. Despite this, citizen complaints of not getting public service on time are heard a lot. Therefore, to maintain and demonstrate integrity, civil servants must deliver public service on time as per the provision by law. Citizens have the right to timely service, yet they did not receive it for various reasons. Sometimes civil servants deliberately do not provide services on time to force citizens to pay extra money (Pokharel et al., 2018). Deliberately delaying to provide public services is a sign of unethical behavior and violation of integrity, and it opens the door for a bribe. Citizens are sometimes forced to pay more money than a service charge, while citizens sometimes voluntarily offer bribes by forming informal settings with civil servants to receive service as quickly as possible (Bhattarai and Gupta, 2022). Whether citizens offer a bribe or civil servants are forced to accept a bribe to receive public service timely undermines civil servants’ integrity. Therefore, civil servants should deliver bribery-free public service on time to maintain integrity.

### 5.4 Hassle-free service

On-time, hassle-free public services for citizens are becoming a prominent agenda. Nowadays, civil servants are under pressure to deliver hassle-free public services. Complicated procedures in service delivery cause citizens to suffer and squander their money and time. Civil servants have a duty, and citizens have the right to get hassle-free public services on time; however, evidence suggests that citizens face difficulties. According to the NNGS 2017/18, more than half (53.9%) of citizens reported that government employees create hassle in service delivery (NASC, 2018). These statistics show that civil servants make a hassle in delivering public services, which has a detrimental impact on their ethics and integrity. The effect of hassle-free public service on the integrity of civil servants is enormous and tremendous. Civil servants who deliver hassle-free public service are more likely to demonstrate a high level of integrity, as confirmed by this study. Therefore, civil servants should deliver hassle-free service on time for maintaining and demonstrating integrity. Providing hassle-free service strengthens civil servants’ ethics and integrity.

To get hassle-free service on time, citizens look for alternative ways to get work done faster because of lengthy procedures and complex administrative structures (Hooda, 2016). Sometimes civil servants inadvertently and intentionally create hassle and complexity for seeking additional incentives from citizens. Citizens pay extra money because the cost of giving a bribe seems more bearable than the delay to them (Hooda, 2016). The citizens are also indirectly forced to seek help from the intermediaries by showing artificial legal and procedural hassles. Citizen, therefore, seeks the assistance of intermediaries to avoid procedural complexities, extra bureaucratic hurdles, or red tape deliberately created by civil servants. Citizens may encounter complex administrative processes if they wish to receive service alone. Adding complexity, more bureaucratic hurdles, and making space for intermediaries violate integrity. Hence, civil servants should inform citizens about all legal administrative procedures proactively and reactively, and empathically assist them in simplifying managerial procedures. It guarantees efficiency in public service, and fosters integrity in civil servants.

### 5.5 Connection/network

The influence of connection/network in public service seems strong in Nepal. Nepali citizens believe that personal connections/networks are vital for work done in government offices. According to NNGS 2017/18, 86.2 percent of citizens agreed that work can be done in any way if one has a connection/network (NASC, 2018). Principally, connections/networks with civil servants should be less meaningful in public service, but it has got more important. Citizens feel that having a connection/network with civil servants allows them to acquire services quickly and conveniently. Civil servants become more responsive, and deliver public services conveniently and effectively in less time if citizens are connected to/networked with them (Bhattarai and Gupta, 2022). If citizens aren’t connected to/network with civil servants, the prospects of receiving public service without difficulty are relatively low.

In Nepal, civil servants favoring citizens within their connection/network are deemed to have their social onus, which is seen as usual in public service, but affects negatively their integrity, as shown by this study. Here, the favorable treatment of citizens inside their connection/network breaches the notion of justice and fairness, which lessens the civil servants’ integrity. Hence, fairness and non-discrimination practices are crucial for maintaining integrity in civil servants. Public officials must be fair in their work, particularly in encounters with the public, and they must never offer undue preference to any group or individual (United Nations, 1996). Fairness and justice are moral and ethical obligations of civil servants that must be met in delivering public services to maintain integrity. The culture of preferential treatment of citizens inside a connection/network should be minimized. It ensures fairness, justness, and equality in public service, and nurtures integrity for civil servants.

### 5.6 Salary

Salary and the integrity of civil servants in public services are inextricably linked. Although it is widely assumed that increased pay is not enough to predict high levels of ethics and integrity in civil servants, this study found that adequate pay fosters integrity. Civil servants having good salaries may endorse ethics and integrity in public service. To a lesser extent, income insufficiency and salary discontent have a detrimental impact on civil servants’ ethical behavior (Meyer-Sahling et al., 2018). Inadequately paid civil servants are more likely to engage in illegal and unethical rent-seeking, whereas well-paid civil servants provide less corrupt and more ethical public services (Van Rijckeghem and Weder, 2001). Increased pay for civil servants is likely to reduce unethical practices such as corruption. Gong and Wu (2012) state that poor civil service salaries have been widely recognized as a pivotal contributor to bribery. Moreover, they noted that when government officials’ salaries are low, but their expectations endure high, they may seek higher remuneration through illegal routes than what is legally allowed, resulting in corruption.

Civil servants indulge in corruption only if they think they are not being paid a fair wage, which can be addressed with higher pay (Mahmood, 2005). Pay is a significant incentive for civil servants not to be corrupt or dishonest (Amundsen and Pinto De Andrade, 2009). Therefore, civil servants should be satisfied with their salaries to retain their ethics and integrity, and deter corruption. According to a survey on civil service management in Nepal, just 39 percent of civil servants are satisfied with their salaries, while most are unsatisfied (Meyer-Sahling et al., 2018). Underpaid civil servants, in general, engage in unethical behavior and corrupt practices to maintain their basic living standards. That could be the fact; scholars such as Van Rijckeghem and Weder (2001), Tanzi (1998), and Hall (2012) have claimed that raising civil servant salaries would help to tackle the corruption problem. Increasing pay may dissuade corruption and improve integrity in civil servants, but it may not be fully assured, because civil servants’ integrity affects by several factors other than salary.

### 5.7 Corruption

In public and academic discourse, the concepts of integrity and corruption are intensely linked (Heywood, 1997; Heywood and Rose, 2014). Corruption is frequently used as an antonym for integrity (Amundsen and Pinto De Andrade, 2009). Corruption is synonymous with unethical behavior or integrity violation (Huberts, 2018). Corruption is a violation of ethical values for personal interest. The violation of ethical values is linked with rising corruption (Shakya, 2009). Principally, civil servants should not seek or accept bribes from the citizens they serve. Evidence, however, showed that civil servants accept/ask bribes for public service. According to the NNGS 2017/18, 10 percent of citizens who obtained public service from government offices had to pay a bribe. Similarly, 13 percent of the citizens reported they paid additional fees other than service fees to receive service (NASC, 2018). Accepting/asking for a bribe for public service is a violation of integrity.

As confirmed by this study, accepting/asking for a bribe negatively affects civil servants’ integrity. Civil servants, who demand bribes in a swap for public service, hollow public service values and violate integrity. There is a legal provision for not paying/asking for a bribe in public services, yet civil servants indirectly pushed citizens to pay a bribe in some cases. Citizens have to pay a bribe if civil servants ask/seek because the state has a monopoly on public services. Civil servants capitalize monopoly of public service and ask for a bribe. Bribery diminishes public services, violates the principle of fairness, and undermines civil servants’ integrity (Hall, 2012). Bribe, in any form, devalues civil servants' public service and ethical behavior. To maintain their integrity, public servants should not receive anything from citizens. Ethical culture is the most effective means to defend against corruption and promotes integrity in civil servants.

## 6 Implication of the study

This study identified the factors affecting the integrity of civil servants in the delivery of public services in Nepal, which can be helpful for policy makers, politicians, and practitioners to pursue better policy and governance approaches in the future. The study has practical implications. It is evident that the integrity of civil servants is positively affected by compliance with rules, responsiveness, timely delivery service, and hassle-free service, while it is negatively affected by civil servants’ prior network/connection with citizens and their asking or receiving a bribe for public service. This implies that if civil servants are compliant (follow the rule), responsive, provide hassle-free services on time, avoid preferential treatment of citizens they know personally, and do not ask for bribes, they will demonstrate a high degree of integrity in the provision of public services. Therefore, concerned authorities, including policy makers, politicians, and practitioners, should consider these factors to ensure and restore civil servants’ integrity in delivering public service. The finding of this study also induces civil servants to be self-motivated and committed to showing a higher degree of integrity in their work performance. It also provides clear guidance for citizens to make civil servants accountable, honest, and trustworthy in their performance. Importantly, it provides insightful evidence to public sector leaders to promote an honest and ethical work culture.

## 7 Conclusion

Civil servants' integrity in delivering public services is the backbone of public sector governance. The government of Nepal has taken several initiatives to practice public service ethos, such as honesty, impartiality, transparency, accountability, diligence, professionalism, and integrity, as part of the organization's culture in the delivery of public service. However, citizens are not fully satisfied, and they criticize civil servants for failing to demonstrate these values in delivering public services. It raises serious concerns about the integrity of civil servants. The integrity of civil servants is complex, as it is impacted by multiple factors such as compliance with rules, responsiveness, timely service delivery, hassle-free service, salary, prior network/connection with citizens and asking for a bribe. These factors, directly and indirectly, affect civil servants' integrity in delivering public service. Therefore, concerned authorities should consider these factors to build and maintain the integrity of civil servants. Demonstrating high integrity in public service is crucial and may also help others be ethical. Cultivating a culture of integrity in the civil service is imperative, but it is not achieved overnight and requires collective action.

## Declarationss

### Author contribution statement

Rajan Khanala: Conceived and designed the experiments; Performed the experiments; Contributed reagents, materials, analysis tools or data; Wrote the paper.

Anil Kumar Gupta: Conceived and designed the experiments; Performed the experiments; Analyzed and interpreted the data; Contributed reagents, materials, analysis tools or data; Wrote the paper.

Prakash C Bhattarai: Analyzed and interpreted the data; Contributed reagents, materials, analysis tools or data; Wrote the paper.

### Funding statement

This research did not receive any specific grant from funding agencies in the public, commercial, or not-for-profit sectors.

### Data availability statement

Data will be made available on request.

### Declaration of interest’s statement

The authors declare no competing interests.

### Additional information

No additional information is available for this paper.
